# Charge-Tunable Polyelectrolytes Enable High-Performance Layer-by-Layer Nanofiltration Membranes for Heavy Metal Ion Removal

**DOI:** 10.3390/membranes16040130

**Published:** 2026-03-31

**Authors:** Fei Jiang, Wenyan Huang, Yifang Mi

**Affiliations:** Key Laboratory of Advanced Textile Materials and Manufacturing Technology and Engineering Research Center for Eco-Dyeing & Finishing of Textiles, Ministry of Education, Zhejiang Sci-Tech University, Hangzhou 310018, China

**Keywords:** layer by layer self assembly, positively charged nanofiltration membrane, heavy metal ions removal, Mannich reaction

## Abstract

Wastewater contamination by toxic heavy metal ions poses a huge threat to ecosystem integrity and human health. Herein, we designed a polyelectrolyte (T-PEI) with a tunable positive charge property to construct a layer-by-layer (LBL) nanofiltration membrane for efficient heavy metal ion removal. The T-PEI was obtained via a Mannich reaction between polyethyleneimine (PEI) and tetrakis (hydroxymethyl) phosphonium chloride (THPC). The introduction of THPC imparted T-PEI with a strong and tunable positive charge, attributed to the quaternary phosphonium groups in THPC. Converting the weakly charged PEI into the strongly charged T-PEI allowed regulation of both T-PEI’s deposition behavior and the electrostatic interactions with sodium polystyrenesulfonate (PSS) during LBL assembly. As a result, after depositing only one bilayer, the positively charged PSS/T-PEI membrane achieved a pore size radius of 0.35 nm, meeting the typical criteria for nanofiltration membranes. Under the optimal preparation conditions, the resultant membranes exhibited a water flux of 38.1 L m^−2^ h^−1^ and high rejections to various heavy metal ions at low operation pressure, such as Cr^3+^ (99.8%), Ni^2+^ (96.1%), Cu^2+^ (92.5%), and Mn^2+^ (90.3%). Additionally, the membrane possessed robust operation stability, along with excellent antifouling/bacterial performance. After cyclic filtration of a lysozyme solution, the flux recovery ratio reached 94.7%. The membrane also exhibited effective bactericidal activity against both *Escherichia coli* (*E. coli*) and *Staphylococcus aureus* (*S. aureus*), with no visible microbial colonies observed. This work highlights the effectiveness of tailoring polyelectrolyte characteristics in enhancing the LBL membrane performance and presents a promising LBL nanofiltration membrane for heavy metal ion removal.

## 1. Introduction

The increasing scarcity of freshwater resources has prompted extensive research into efficient and sustainable water purification technologies [1,2,3]. Statistics from the World Health Organization (WHO) indicate that nearly a quarter of the global population experiences water shortage [4,5]. Compounding this issue, rapid industrialization has introduced serious environmental concerns, including wastewater contamination by toxic heavy metal ions such as Co^2+^, Cr^3+^, Mn^2+^, Ni^2+^, and Cu^2+^ [6,7,8]. These heavy metal ions present substantial environmental and biological risks owing to their high solubility in water and strong persistence, which hinder natural degradation and promote accumulation in living organisms [9,10]. This bioaccumulation threatens both ecosystem integrity and human health, underscoring the critical need for effective mitigation strategies. The WHO specifies the concentration limits for Cr^3+^, Mn^2+^, and Ni^2+^ in safe drinking water as 0.05 mg L^−1^, 0.08 mg L^−1^, and 0.1 mg L^−1^, respectively [6]. However, the content of heavy metal ions in industrial wastewater usually surpasses the WHO-recommended levels. Consequently, developing reliable methods to remove heavy metal ions from wastewater has become an urgent priority.

Numerous well-established methods, such as ion exchange [11], precipitation [12], adsorption [13], and electrochemical treatment [14], have been applied to eliminate heavy metal ions in wastewater. However, these methods are hindered by the generation of hazardous sludge or liquid waste, which poses risks to both the environment and human health. Moreover, their inability to ensure long-term operational stability further limits their overall efficiency and practical applicability. Nanofiltration membranes feature the advantages of energy efficiency, environmental benefits, and ease of operation and have received extensive attention in the realm of wastewater treatment [15,16]. For example, An et al. converted a negatively charged polyamide nanofiltration membrane into a positively charged one via chemical grafting of PEI. The resulting membrane exhibited a pure water permeance of 7.2 L m^−2^ h^−1^ bar^−1^ and a high rejection rate of >95% for copper ions [17]. Hosseini et al. fabricated a polyethersulfone-based nanofiltration membrane by incorporating CoFe_2_O_4_/CuO nanoparticles. The incorporation of these nanoparticles enhanced the membrane’s hydrophilicity and porosity, thereby endowing it with a water flux of 34.5 L m^−2^ h^−1^ bar^−1^ and a heavy metal ion rejection of over 88% [18]. Gong et al. designed a novel pyridine-diamine monomer to regulate the charges and microstructures of the resulting interfacial polymerization membrane. The membrane demonstrates a notable water permeance of ~7.67 L m^−2^ h^−1^ bar^−1^, along with superior rejection (>98%) toward heavy metal ions such as Zn^2+^, Mn^2+^, and Cu^2+^ [19]. These findings indicate that fine-tailoring the membrane microstructure and positive surface charge enables the efficient removal of heavy metal ions through the synergistic action of steric hindrance and the Donnan effect [20,21,22]. These requirements call for highly tunable membranes, a key advantage offered by layer-by-layer (LBL) nanofiltration membranes [23]. The LBL assembly process entails the alternate coating of cationic polyelectrolytes and anionic polyelectrolytes onto a substrate for multiple cycles [24,25]. Key process parameters, such as polyelectrolyte concentration, polyelectrolyte types, salt concentration, solution pH, and number of bilayers, can be tailored. This offers a pathway to flexibly control the surface charge density and pore size distribution of the resulting nanofiltration membrane. For example, Scheepers et al. revealed that increasing the salt concentration in the polyelectrolyte solution would result in a greater extent of extrinsic charge compensation, forming a thicker LBL membrane [26]. Regenspurg et al. found that tuning of the polyelectrolyte molecular weight allows for the adjustment of the membrane’s surface charge (positive/negative) and its pore size [27]. Although numerous LBL nanofiltration membranes have been designed for the selective separation of ions, including heavy metal ion removal [28,29,30], this technique still suffers from a tedious and time-consuming process. Due to the limited adsorption amount of polyelectrolyte, multiple cycles of deposition, rinsing, and drying are typically required to avoid membrane defects.

Given that the LBL self-assembly process is driven by electrostatic interactions, the charge density of polyelectrolytes is the paramount parameter governing the membrane formation process [31,32]. The strength of polyelectrolyte charge not only influences the intensity of electrostatic interactions between ion pairs but also affects the molecular conformation of the polyelectrolytes, thereby altering their deposition behavior [30,32,33]. This, in turn, impacts the pore structure and surface charge properties of the resulting LBL nanofiltration membranes. Therefore, adjusting the charge properties of polyelectrolytes effectively regulates their deposition behavior, providing a potential for improving preparation efficiency.

Herein, we designed a strong positively charged polyelectrolyte (denoted as T-PEI) to construct an LBL nanofiltration membrane with only one bilayer for efficient heavy metal ion removal. The T-PEI was obtained through the Mannich reaction between PEI and THPC. THPC is a molecule containing a quaternary phosphonium group, whose four hydroxyl groups enable it to react with primary and secondary amines through the Mannich reaction [34,35,36]. The charge properties of T-PEI could be easily regulated via tuning the concentration of THPC, which can effectively regulate the deposition behavior of T-PEI. Therefore, by assembling only one bilayer on the substrate, the resultant nanofiltration membrane (PSS/T-PEI) exhibited high efficiency in removing heavy metal ions, as well as excellent antifouling and antibacterial properties.

## 2. Experimental Section

### 2.1. Materials and Chemicals

PSS (Mw = 70,000 Da), PEI (Mw = 10,000 Da), polyethylene glycol (PEG, Mw = 1000, 800, 600, 400, and 200 Da), chromium chloride hexahydrate (CrCl_3_·6H_2_O, ≥98%), nickel chloride hexahydrate (NiCl_2_·6H_2_O, ≥97%), cobalt chloride hexahydrate (CoCl_2_·6H_2_O, ≥97%), copper chloride (CuCl_2_, ≥98%), manganese chloride (MnCl_2_, ≥98%), magnesium chloride (MgCl_2_), barium chloride dihydrate (BaCl_2_·2H_2_O, ≥99.5%), calcium chloride (CaCl_2_, ≥96%), potassium chloride (KCl, ≥99.5%), sodium chloride (NaCl, ≥99.5%), and lysozyme (LYZ) (≥20,000 U·mg^−1^) were supplied by Aladdin Reagent Co., Ltd. (Shanghai, China). THPC (75% in water) was provided by Macklin Biochemical Technology Co., Ltd. (Shanghai, China). *E. coli* and *S. aureus* were provided by Shanghai Luwei Technology Co., Ltd. (Shanghai, China). Polysulfone (PSF) substrate with a molecular weight cut-off (MWCO) of 20 kDa was supplied by Zhongke Ruiyang Membrane Technology Co., Ltd. (Beijing, China). Deionized water was used in all experiments.

### 2.2. Fabrication of PSS/T-PEI Membranes via LBL Assembly

**Figure 1:**
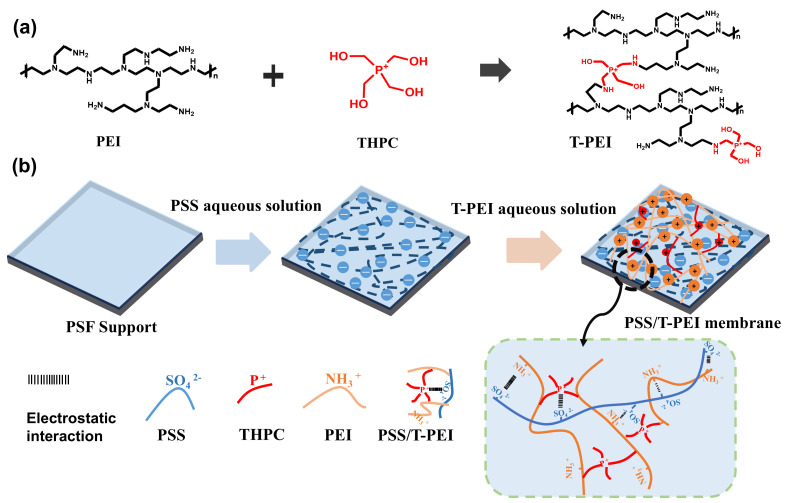
Schematic diagram of (**a**) Mannich reaction between PEI and THPC and (**b**) PSS/T-PEI LBL nanofiltration membrane.

The polycation T-PEI was synthesized beforehand through the Mannich reaction between PEI and THPC, and the PSS/T-PEI membrane was prepared through LBL self-assembly of PSS and T-PEI. Specifically, a certain amount of THPC was added in 0.9 wt% PEI aqueous solution to generate the Mannich reaction (Figure 1a and Table 1), and the mixture (T_x_-PEI, where x represented the THPC concentration) was stirred at 400 rpm for 30 min before use. The PSF substrate was exposed to 20 mL of a 0.2 g L^−1^ PSS solution for 15 min, which contained 0.05 M NaCl. Subsequently, the substrate was rinsed with deionized water three times to remove loosely adhered PSS before the next deposition step. Then, the PSS-coated substrate was soaked in the 20 mL T_x_-PEI aqueous solution for 2 min and rinsed with deionized water three times. A bilayer was then obtained. Finally, the assembled PSS/Tx-PEI membrane was dried at 50 °C for 15 min. Membranes prepared with different THPC concentrations (0.3, 0.4, 0.5, 0.6, and 0.7 wt%) were denoted as PSS/T_x_-PEI, where x represented the THPC concentration used in T-PEI synthesis. For comparison, the membrane prepared via the LBL method between PEI and PSS was denoted as PSS/PEI.

### 2.3. Characterizations

The membranes’ chemical compositions and structures were analyzed by attenuated total reflectance–Fourier-transform infrared spectroscopy (ATR-FTIR, Nicolet Avatar 370, Madison, WI, USA) and X-ray photoelectron spectroscopy (XPS, K-Alpha, Thermo Scientific, Waltham, MA, USA), respectively. The surface morphologies and roughness were confirmed by scanning electron microscopy (SEM, GeminiSEM 500, Zeiss, Oberkochen, Germany) and atomic force microscopy (AFM, XE-100E, Park Systems, Suwon, Republic of Korea), respectively. The membrane hydrophilicity was measured by a contact angle meter (Data physics Instruments GmbH, Filderstadt, Germany). An electrokinetic analyzer (SurPASS Anton Paar GmbH, Graz, Austria) was used to confirm membrane surface charge at pH 5.0. The diffusion coefficient of T-PEI in solutions prepared with various THPC concentrations was measured by dynamic light scattering [37].

### 2.4. Nanofiltration Performance

The nanofiltration performances of the PSS/T-PEI membranes were validated by a cross-flow nanofiltration system at 0.6 MPa. The water flux (J) and salt rejection (R) were calculated via the following equations:

(1) $$J=\frac{V}{S\times t}$$

(2) $$R\left(\%\right)=\frac{C_{f}-C_{p}}{C_{f}}\times 100$$

where S (m^2^) represents the effective membrane area, V (L) is the permeate volume collected over a time interval t (h), and C_f_ and C_p_ are the solute concentrations in the feed and permeate solutions, respectively. The solute concentration of a single salt solution was confirmed by a conductivity meter (DDSJ-308A, Leici, Shanghai, China), and the solute concentration of a mixture salt solution was measured by inductively coupled plasma optical emission spectrometry (ICP-OES, Agilent 5110, Santa Clara, CA, USA). The MWCO of the PSS/T-PEI membranes was determined by filtering PEG aqueous solutions (0.2 g·L^−1^), and the concentration of PEGs was characterized by a Shimadzu total organic carbon analyzer.

### 2.5. Antifouling and Antibacterial Performance

The antifouling performance was evaluated through cyclic fouling tests. Using a cross-flow filtration system, the pressure was first stabilized at 0.6 MPa, after which the membrane pure water flux (J_0_) was measured over a 2 h period. The feed was then switched to a 0.1 g·L^−1^ LYZ solution for 3 h, and the water flux (J_f_) was recorded. Then, the fouled membrane was washed with deionized water for 2 h, and its recovered pure water flux (J_1_) was measured. Then, a second identical fouling cycle was conducted. The water flux at the end of the second fouling cycle is denoted as J_h_, and the pure water flux after the second cleaning process is defined as J_2_. The flux recovery rate (FRR), irreversible fouling ratio (FR_ir_), and reversible fouling ratio (FR_r_) were calculated using Equations (3)–(5), respectively.

(3) $$FRR\left(\%\right)=\frac{J_{2}}{J_{0}}\times 100$$

(4) $$FR_{ir}\left(\%\right)=\frac{J_{0}-J_{2}}{J_{0}}\times 100$$

(5) $$FR_{r}\left(\%\right)=\frac{J_{2}-J_{h}}{J_{0}}\times 100$$

The antibacterial activity of the PSS/T-PEI membrane was evaluated using *E. coli* and *S. aureus* as model biological pollutants. The membrane (1 cm^2^) was first sterilized under UV irradiation and then immersed in bacterial suspensions with a concentration of 10^7^ CFU·mL^−1^ for each strain. To promote adequate interaction between bacteria and the membrane, the inoculated samples were placed in a constant-temperature shaker at 37 °C for 4 h. After incubation, the membranes were carefully removed and rinsed lightly with deionized water. The collected rinse eluent was diluted by a factor of 400, and 50 µL of the diluted solution was plated onto solid agar. The colony-forming units were counted to evaluate the antimicrobial activity of the membrane after incubation at 37 °C for 18 h. It should be noted that the UV light exposure did not alter the membrane properties (Figure S1).

## 3. Results and Discussion

**Figure 2:**
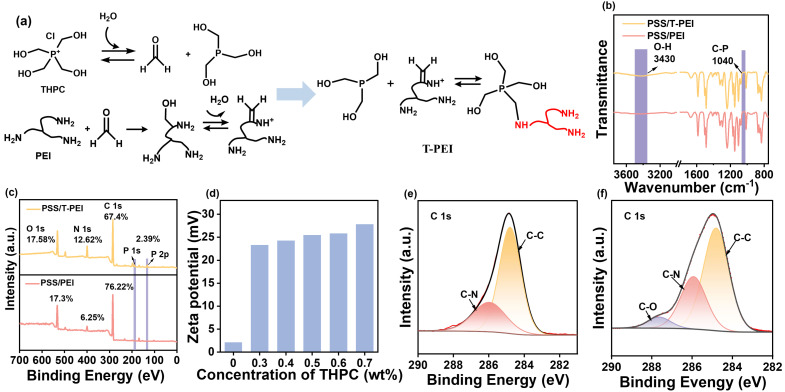
(**a**) The mechanism of reaction between PEI and THPC; (**b**) ATR-FTIR; (**c**) XPS spectra of PSS/PEI and PSS/T-PEI membranes, respectively; (**d**) Zeta potential of T_x_-PEI with different THPC concentrations; (**e**,**f**) high-resolution C 1 s spectra of PSS/PEI and PSS/T-PEI membranes, respectively.

The membrane was fabricated using the LBL assembly technique, employing PSS as the polyanion and the T-PEI as the polycation. The T-PEI was synthesized via the Mannich reaction between PEI and THPC. During the reaction, formaldehyde is generated in situ from THPC decomposition. This formaldehyde subsequently reacts with the primary amine groups of PEI to form an immonium ion intermediate. Finally, a THPC-derived species reacts with the immonium ion, completing the process through hydroxymethyl arm substitution [35], as illustrated in Figure 2a. This reaction transforms the weak polyelectrolyte PEI into the strong polyelectrolyte T-PEI, and the charge properties of T-PEI could be adjusted through the concentration of THPC. The chemical structures and element contents of membranes were characterized by ATR-FTIR and XPS, respectively. Compared with the PSS/PEI membrane, a new characteristic peak at 1040 cm^−1^, corresponding to the C–P bond in THPC [38], was observed in the PSS/T-PEI membrane (Figure 2b). Additionally, the intensity of the O-H stretching band at ~3362 cm^−1^ increased, likely due to the incorporation of the -OH groups during the Mannich reaction [39]. XPS analysis further confirmed the presence of THPC-PEI structures, as phosphorus (P) was detected only in the PSS/T-PEI membrane (Figure 2c). Meanwhile, the PSS/T-PEI membrane possessed higher N content, indicating that a greater proportion of T-PEI was complexed with PSS compared to the PSS/PEI membrane. The positively charged P^+^ groups derived from THPC substantially enhanced the overall positive charge density of the T-PEI (Figure 2d), thereby promoting its electrostatic complexation with PSS. As shown in Figure 2e,f, the high-resolution C 1 s spectra of both membranes display characteristic peaks representing C–C (284.8 eV) and C–N (285.9 eV) [40,41]. A new peak emerged at 287.6 eV in the PSS/T-PEI spectrum, which was attributed to C–O groups from THPC [42]. Consistent with the reaction pathway illustrated in Figure 2a, the Mannich reaction proceeds via an iminium ion intermediate, forming additional C–N covalent linkages. This is further supported by the increase in C–N content from 29.4% in the PSS/PEI membrane to 31.9% in the PSS/T-PEI membrane, confirming the successful incorporation of THPC through amine-bond formation.

**Figure 3:**
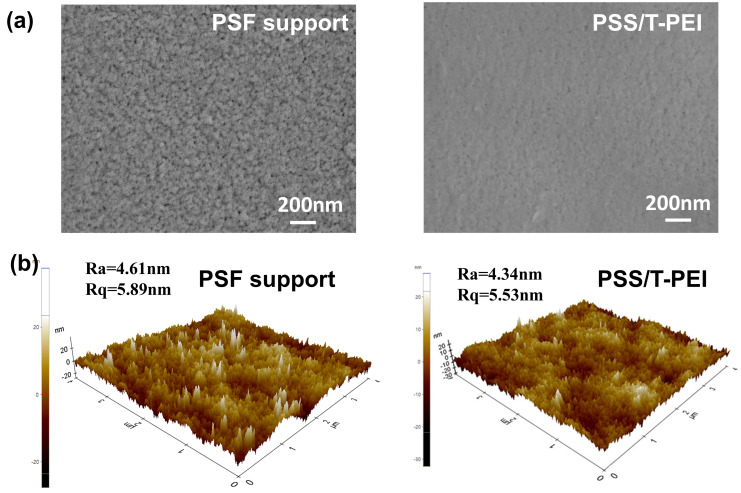
(**a**) SEM and (**b**) AFM images of PSF substrate and PSS/T-PEI membrane.

Figure 3a presents the surface morphology of the PSF substrate and PSS/T-PEI membrane. The PSF substrate exhibited a distinct porous structure, and the porous structure of the PSF substrate remained clearly visible in the PSS/PEI one-bilayer-assembled membrane (Figure S2). In contrast, after only one bilayer self-assembly of PSS and T-PEI, a uniform, continuous PSS/T-PEI membrane was formed on the PSF substrate. This further manifested that the resulting T-PEI deposited more readily on the surface of the PSF substrate compared to PEI. The deposition of T-PEI effectively covered the porous PSF substrate, resulting in a smoother surface morphology. Consequently, the PSS/T-PEI membrane exhibited a lower surface roughness compared to the PSF substrate (Figure 3b). All these results collectively demonstrated the successful occurrence of the Mannich reaction between THPC and PEI, and the resulting T-PEI was assembled onto the PSS-deposited layer via electrostatic interactions.

**Figure 4:**
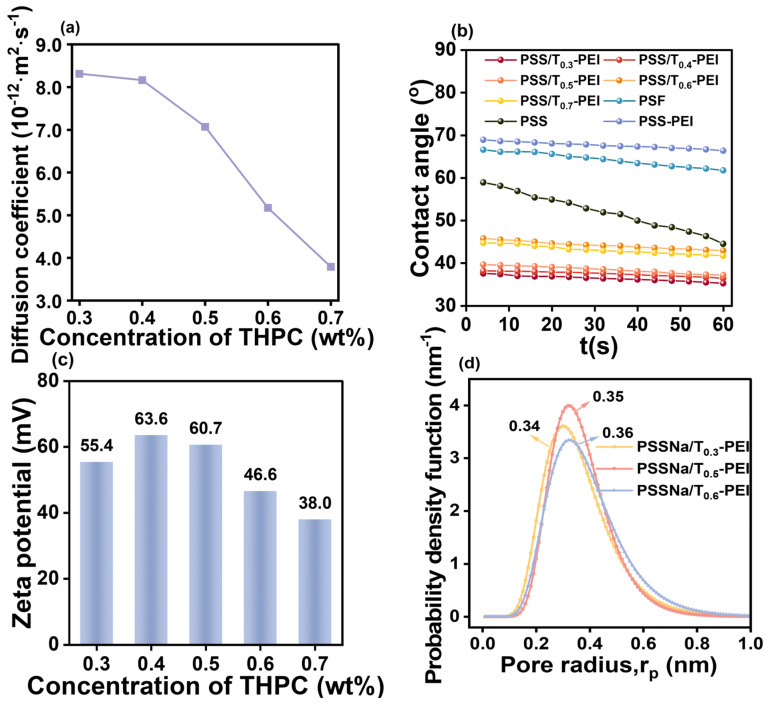
(**a**) Diffusion coefficient of T-PEI with various THPC concentrations; (**b**) water contact angles of PSS/T-PEI membranes with different THPC concentrations, PSS-deposited membrane, PSS/PEI membrane, and PSF substrate, respectively. (**c**) Effect of THPC concentrations on the surface charge of PSS/T-PEI membrane (pH 5); (**d**) pore size distributions of PSS/T_0.3_-PEI, PSS/T_0.5_-PEI, and PSS/T_0.6_-PEI membranes, respectively.

The membrane properties of the PSS/T-PEI membrane could be tuned by adjusting the THPC concentration. As shown in Table 2, the N content of the PSS/T-PEI membranes was all higher than that of PSS/PEI (6.25 at%). This indicated that T-PEI exhibited higher assembly efficiency than PEI under the same preparation conditions. Meanwhile, the N content of the PSS/T-PEI membrane decreased with the increasing THPC concentration. To explain this phenomenon, the diffusion coefficient and Zeta potential of T-PEI with various THPC concentrations were measured (Figure 4a and Figure 2d). The decreased diffusion coefficient of T-PEI indicated that its deposition rate on the PSS layer slowed as the THPC concentration increased [43]. Meanwhile, as shown in Figure S1, a higher THPC concentration yielded T-PEI with a higher charge density, meaning it possessed more positively charged groups. Consequently, less T-PEI adsorption was required to compensate for the negative charges of the PSS layer pre-adsorbed on the PSF substrate [30]. The reduced diffusion rate and enhanced positive charge of T-PEI together resulted in a decrease in the deposition amount of T-PEI as the THPC concentration increased. Consequently, a decrease in N content on the PSS/T-PEI membrane was observed with increasing THPC concentration. Benefiting from the excellent water-binding capacity of THPC [44,45], the PSS/T-PEI membranes exhibited improved surface hydrophilicity (Figure 4b); the water contact angles remained significantly lower than those of the PSS-deposited layer and the PSS/PEI membrane. Meanwhile, all PSS/T-PEI membrane surfaces featured a strong positive charge after only one bilayer assembly (Figure 4c). The positive charge on the membrane surface initially increased and then decreased with increasing THPC concentration. As the THPC concentration increases, the positive charge of T-PEI gradually strengthens (Figure 2d), which is theoretically expected to enhance the positive charge on the PSS/T-PEI nanofiltration membrane surface. However, as shown in Figure 4a, the diffusion rate of Tx-PEI decreases with increasing THPC concentration, leading to a reduction in its adsorption amount on the PSS deposition layer, thereby weakening the surface positive charge of the membrane. It is proposed that the interplay between these two opposing effects governs the observed non-monotonic trend, wherein the surface positive charge initially increases and subsequently decreases with rising THPC concentration. The pore size distributions of PSS/T-PEI membranes with different THPC concentrations are illustrated in Figure 4d. The pore size radii of PSS/T_0.3_-PEI, PSS/T_0.5_-PEI, and PSS/T_0.6_-PEI were 0.34, 0.35, and 0.36 nm, respectively. Typically, effective nanofiltration separation via LBL assembly necessitates the deposition of multiple polyelectrolyte layers. However, the PSS/T-PEI membrane prepared with only one bilayer assembly already met the pore size criteria expected for a nanofiltration membrane. The rational design of T-PEI can effectively enhance the self-assembly efficiency, enabling the successful fabrication of nanofiltration membranes with only one bilayer assembled. This fully demonstrated the feasibility and effectiveness of this strategy. For comparison, the PSS/PEI membrane exhibited a CoCl_2_ rejection of only about 10% (Figure S3), which was far below the requirement for nanofiltration desalination. These results demonstrated that the integrated structure of the PSS/T-PEI membrane was not merely due to the high polyelectrolyte concentration (0.9 wt%), but more critically, to the introduction of THPC. On the one hand, THPC significantly enhances the positive charge of the T-PEI, strengthening the charge-sieving effect against target ions. On the other hand, THPC acts as a cross-linker for PEI, improving the compactness and structural stability of the membrane. The synergistic effect of these two factors enables T-PEI to deposit effectively, thereby endowing the PSS/T-PEI membrane with efficient nanofiltration separation. Consequently, this well-structured bilayer membrane is expected to effectively remove heavy metal ions.

**Figure 5:**
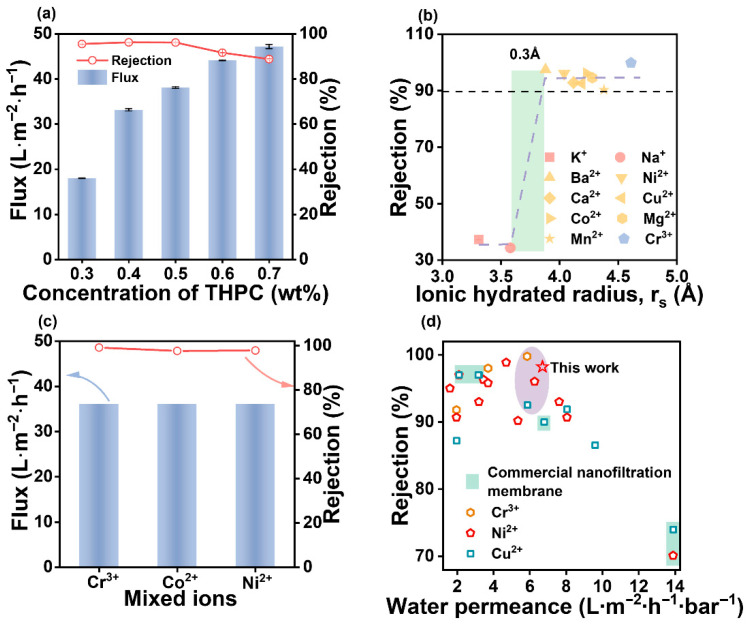
(**a**) Effect of THPC concentrations on the PSS/T-PEI membrane separation performance (salt concentration: 0.5 g L^−1^); (**b**) the ion rejections of PSS/T_0.5_-PEI membranes (salt concentration: 0.5 g L^−1^); (**c**) ion rejections of PSS/T_0.5_-PEI membrane when separating mixture salt solution (the mixture solution: CrCl_3_ 0.25 g L^−1^, CoCl_2_ 0.25 g L^−1^, NiCl_2_ 0.25 g L^−1^); (**d**) comprehensive comparison of PSS/T0.5-PEI membrane with state-of-the-art nanofiltration membranes.

The separation performance of the PSS/T-PEI membrane prepared with various THPC concentrations was investigated with a 0.5 g L^−1^ CoCl_2_ aqueous solution. As displayed in Figure 5a, with the increasing of THPC concentrations, the water flux increased from 17.9 to 47.2 L m^−2^ h^−1^; meanwhile, the CoCl_2_ rejection slightly increased from 95.5% to 96.1% and then decreased to 88.8%. As discussed above, the deposition amount of T-PEI decreases with increasing THPC concentration, leading to a gradual increase in membrane pore size (Figure 4d) and consequently a rise in water flux. When the THPC concentration increased to 0.5 wt%, the enhanced surface positive charge contributed to the improved CoCl_2_ rejection. When the THPC concentration was further increased, the adsorption amount of T-PEI decreased, resulting in reduced surface positive charge and a looser membrane structure, which led to a decline in CoCl_2_ rejection. In summary, 0.5 wt% was identified as the optimal THPC concentration, at which the membrane achieved the best overall separation performance, with a water flux of 38.1 L m^−2^ h^−1^ and a CoCl_2_ rejection of 96.1%. Therefore, the PSS/T_0.5_-PEI membrane was selected for further investigation into its heavy metal ion removal performance.

Moreover, the rejection of the PSS/T_0.5_-PEI membrane to various salts is displayed in Figure 5b. The ion rejection sequence of the PSS/T_0.5_-PEI membrane was: Cr^3+^ (99.8%) > Ba^2+^ (97.4%) > Co^2+^ (96.1%) > Ni^2+^ (96.1%) > Mg^2+^ (94.5%) > Ca^2+^ (92.9%) > Cu^2+^ (92.5%) > Mn^2+^ (90.3%) > K^+^ (37.2%) > Na^+^ (34.2%). The PSS/T_0.5_-PEI membrane could effectively reject high-valent cationic ions larger than 3.86 Å (rejection > 90%), while allowing small cations (K^+^, Na^+^) to pass (rejection < 40%). Meanwhile, a sharp rejection transition (0.3 Å) was observed in the rejection curves of the PSS/T_0.5_-PEI membrane, indicating superior ion selectivity. The PSS/T_0.5_-PEI membrane was positively charged under the test conditions (pH 5), and there was a strong electrostatic repulsion between the membrane surface and high-valent cationic ions. Additionally, the hydrated radii of these high-valent cationic ions were larger than the pore size radius of the PSS/T_0.5_-PEI membrane (0.35 nm). Thus, the high rejection of high-valent cationic ions of the PSS/T_0.5_-PEI membrane was dominated by the synergistic effect of steric hindrance and the Donnan effect.

Furthermore, when applied to a mixed heavy metal ion solution, the water flux of the PSS/T_0.5_-PEI membrane was maintained at 36.4 L m^−2^ h^−1^. The rejections in the mixed solution were 99.1% for Cr^3+^, 97.8% for Ni^2+^, and 97.6% for Co^2+^. The high rejections can be attributed to the synergistic effect of the membrane’s positive charge and dense structure. These results demonstrate the strong potential of the PSS/T_0.5_-PEI membrane for practical wastewater treatment, given its effectiveness in removing diverse heavy metal ions. A more comprehensive comparison of the PSS/T_0.5_-PEI membrane with state-of-the-art nanofiltration membranes is presented in Figure 5d and Table S1. The PSS/T_0.5_-PEI membrane exhibited heavy metal ion removal performance comparable to that of reported interfacial polymerization membranes. In contrast to other LBL nanofiltration membranes, the PSS/T_0.5_-PEI membrane not only exhibited higher water permeance and ion rejections but also featured a simpler fabrication process, requiring only the assembly of a bilayer to effectively remove heavy metal ions. Furthermore, the PSS/T_0.5_-PEI membrane outperformed some commercial nanofiltration membranes in heavy metal ion removal. Importantly, this superior performance was achieved at low operating pressures, underscoring its energy efficiency and practical applicability.

**Figure 6:**
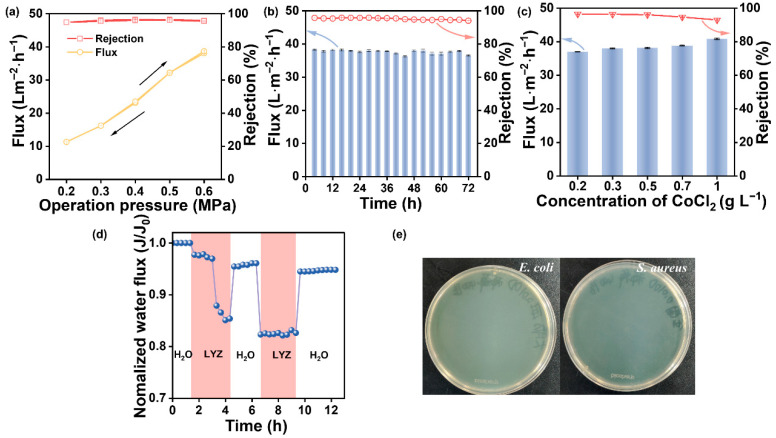
Effects of (**a**) operation pressure, (**b**) operation time, and (**c**) CoCl_2_ concentrations on the separation performance of PSS/T_0.5_-PEI membrane; (**d**) antifouling performance and (**e**) antibacterial performance of PSS/T_0.5_-PEI membrane.

In addition to excellent separation performance for heavy metal ion removal, the operational stability was also essential to meet the critical requirements for practical deployment. Figure 6a shows the effect of operation pressure on PSS/T_0.5_-PEI membrane separation performance. The water flux of the PSS/T_0.5_-PEI membrane increased linearly from 11.3 to 38.1 L m^−2^ h^−1^, and the rejection of CoCl_2_ remained stable when the operation pressure increased from 0.2 to 0.6 MPa. Moreover, the PSS/T_0.5_-PEI membrane exhibited reversible separation performance in the cyclic test, demonstrating that the PSS/T_0.5_-PEI membrane possessed structural stability capable of withstanding changes in pressure [46]. The long-term stability of the PSS/T_0.5_-PEI membrane was also investigated (Figure 6b). During the continuous operation over 72 h, the water flux and CoCl_2_ rejection of the PSS/T_0.5_-PEI membrane were maintained at ~35 L m^−2^ h^−1^ and ~96%. Increasing the CoCl_2_ concentration from 0.2 to 1.0 g L^−1^ led to a slight rise in the water flux of the PSS/T_0.5_-PEI membrane from 37.0 to 40.8 L m^−2^ h^−1^, which was accompanied by a decrease in CoCl_2_ rejection from 96.5% to 93.0%. This was because the increasing salt concentration led to competition between salt ions and the paired polyelectrolyte units, resulting in the dissociation of the polyelectrolyte complex [47]. However, the membrane retained a CoCl_2_ rejection as high as 93%, highlighting its excellent potential in the field of heavy metal ion separation.

Apart from separation performance, antifouling and antibacterial capabilities are also essential for the effective long-term operation of membranes in wastewater treatment. Herein, the antifouling properties of the PSS/T_0.5_-PEI membrane were evaluated by using LYZ as the model foulant. As shown in Figure 6d, during the fouling process, the water flux of the PSS/T_0.5_-PEI membrane decreased, and then the water flux recovered after the cleaning process. The FRR, FRir, and FRr values of the PSS/T_0.5_-PEI membrane were 94.7%, 5.3%, and 12.1%, respectively. The high FRR value after two cycle tests manifested the excellent antifouling properties of the PSS/T_0.5_-PEI membrane. The hydration layer on the hydrophilic membrane surface served as a barrier, hindering direct contact between LYZ foulants and the membrane surface [48,49]. Furthermore, strong electrostatic repulsion between the membrane surface and LYZ also contributed to the reduced foulant deposition [50,51]. Together, these mechanisms endowed the PSS/T_0.5_-PEI membrane with excellent antifouling performance.

Subsequently, the antibacterial properties of the PSS/T_0.5_-PEI membrane against *E. coli* and *S. aureus* were assessed via the plate count method. As shown in Figure 6e, the PSS/T_0.5_-PEI membrane demonstrated effective bactericidal activity against both Gram-negative (*E. coli*) and Gram-positive (*S. aureus*) bacteria, and no obvious microbial colonies were observed. The antibacterial property stems from dual mechanisms. First, the hydrophilic membrane surface prevents bacterial deposition [52]. Second, the chains of PEI can penetrate and disrupt the structural integrity of the bacterial cell wall, ultimately leading to cell death [53]. Additionally, the incorporated quaternary phosphonium salts (THPC, a known antibacterial agent for water systems) actively disrupt microbial cell walls [54]. This combination of passive repulsion and active killing enables the membrane to achieve robust antibacterial performance. Additionally, the PSS/T_0.5_-PEI membrane also exhibited good antibacterial performance during the dynamic antibacterial experiment, with an FRR that could be 90.6% (Figure S5).

## 4. Conclusions

In this work, the polyelectrolyte T-PEI with tunable positive charge was successfully designed and utilized as a polycation to fabricate nanofiltration membranes via the LBL method for efficient removal of heavy metal ions. The positive charge property and deposition amount of T-PEI could be easily tuned by adjusting THPC concentrations, thereby affecting the pore structure and surface positive charge properties of the resultant membrane. After depositing only one bilayer, the optimal PSS/T_0.5_-PEI membrane achieved a water flux of 38.1 L m^−2^ h^−1^ with a high rejection to CoCl_2_ (96.1%). Moreover, when tested with a mixed ion solution, the PSS/T_0.5_-PEI membrane showed high removal efficiency, and the ion rejections were 99.1% for Cr^3+^, 97.8% for Ni^2+^, and 97.6% for Co^2+^. Additionally, the PSS/T_0.5_-PEI membrane exhibited long-term stability, antifouling, and antibacterial performance, highlighting its potential in the treatment of wastewater containing heavy metal ions. This work underlines the effectiveness of tailoring polyelectrolyte characteristics in enhancing the LBL membrane performance and presents a promising LBL nanofiltration membrane for heavy metal ion removal.

## Figures and Tables

**Table 1:** Preparation condition of Tx-PEI.

Name	Concentration	
	PEI (wt%)	THPC (wt%)
T_0.3_-PEI	0.9	0.3
T_0.4_-PEI	0.9	0.4
T_0.5_-PEI	0.9	0.5
T_0.6_-PEI	0.9	0.6
T_0.7_-PEI	0.9	0.7

**Table 2:** Chemical compositions of membrane surfaces.

Membrane	Atomic Concentration (at%)			
	C	N	O	P
PSS/PEI	76.22	6.25	17.3	0
PSS/T_0.3_-PEI	70.46	13.68	14.39	1.47
PSS/T_0.4_-PEI	70.38	12.37	15.74	1.50
PSS/T_0.5_-PEI	67.40	12.62	17.58	2.39
PSS/T_0.6_-PEI	71.79	11.18	15.80	1.23
PSS/T_0.7_-PEI	74.32	10.01	14.83	0.84

## Data Availability

The original contributions presented in this study are included in the article/Supplementary Materials. Further inquiries can be directed to the corresponding author.

## Supplementary Materials

The following supporting information can be downloaded at: https://www.mdpi.com/article/10.3390/membranes16040130/s1, Figure S1: (a) separation performance (test condition: 1 g L^−1^ CoCl_2_ at 25 °C, 0.6 MPa) and (b) ATR-FTIR spectra of PSS/T_0.5_-PEI membrane before and after UV light exposure; Figure S2: SEM image of PSS/PEI membrane; Figure S3: Separation performance of PSS/PEI and PSS/T-PEI membranes when testing with 0.5 g L^−1^ CoCl_2_ aqueous solution; Figure S4: SEM image of PSS/T_0.5_-PEI membrane before fouling, after fouling, and after cleaning; Figure S5: Dynamic antibacterial results for the PSS/T_0.5_-PEI membrane (*E. coli*, 10^8^ CFU·L^−1^); Table S1: Comprehensive comparison of PSS/T_0.5_-PEI membrane with state-of-the-art nanofiltration membranes reported in literature and commercial nanofiltration membranes. References [1,28,29,55,56,57,58,59,60,61,62,63,64,65,66,67,68,69,70] are cited in Supplementary Materials.
