# Supplementary material for: Charge-Tunable Polyelectrolytes Enable High-Performance Layer-by-Layer Nanofiltration Membranes for Heavy Metal Ion Removal
Source: Membranes (Basel). 2026 Mar 31;16(4):130. doi: 10.3390/membranes16040130 (PMC13117816; doi:10.3390/membranes16040130)
Supplement: Supplementary file 1 [file membranes-16-00130-s001.zip › membranes-4196916 Supplementary.pdf]

Charge-Tunable Polyelectrolytes Enable High-Performance Layer-by-Layer Nanofiltration Membranes for Heavy Metal Ion Removal

Fei Jiang, Wenyan Huang, Yifang Mi*

Key Laboratory of Advanced Textile Materials and Manufacturing Technology and Engineering Research Center for Eco-Dyeing & Finishing of Textiles, Ministry of Education, Zhejiang Sci-Tech University, Hangzhou 310018, China

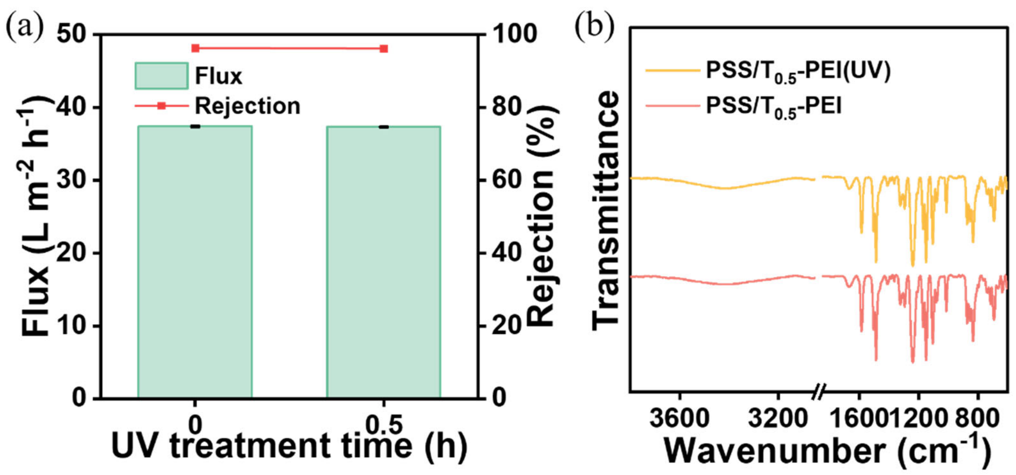

Figure S1. (a) separation performance (test condition: 1 g L^{-1} $CoCl_2$ at 25 ºC, 0.6 MPa) and (b) ATR-FTIR spectra of PSS/$T_{0.5}$-PEI membrane before and after UV light exposure

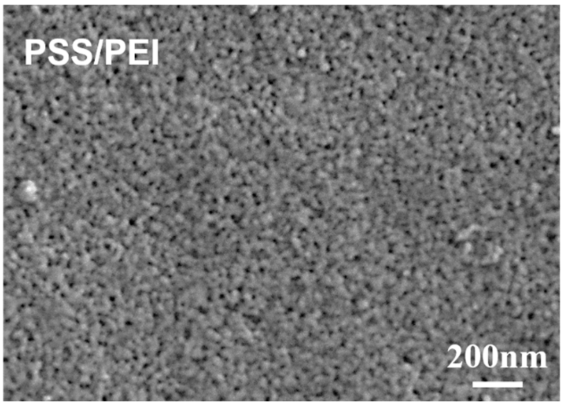

Figure S2. SEM image of PSS/PEI membrane.

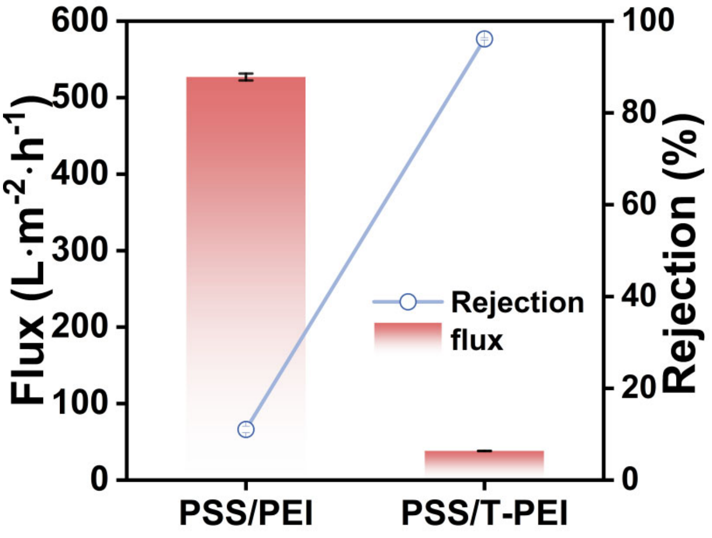

Figure S3. Separation performance of PSS/PEI and PSS/T-PEI membranes when testing with 0.5 g L^{-1} $CoCl_2$ aqueous solution.

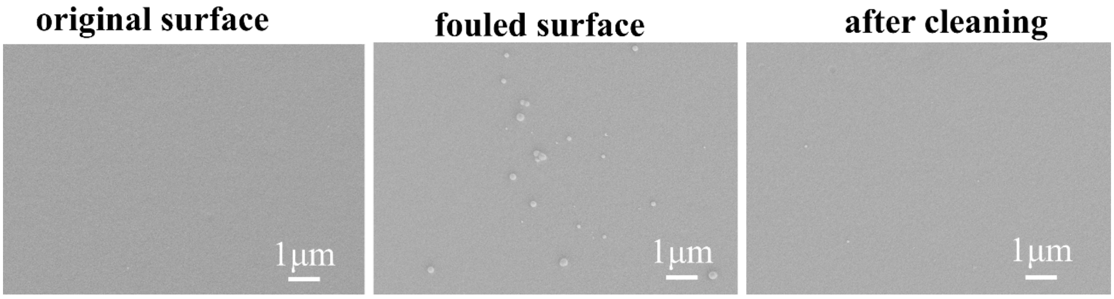

Figure S4. SEM image of PSS/$T_{0.5}$-PEI membrane before fouling, after fouling, and after cleaning.

Table S1. Comprehensive comparison of PSS/$T_{0.5}$-PEI membrane with state-of-the-art nanofiltration membranes reported in literature and commercial nanofiltration membranes.

Membrane	Water permeance ($L\ m^{-2}\ h^{-1}\ bar^{-1}$)	Solute	Concentration (ppm)	Rejection (%)	Method *	Ref
CS-P/T-PEI	10.9	Ni^{2+}	500	93.3	IP	[1]
		Mn^{2+}	500	92.8		
		Cu^{2+}	500	91.0		
M−PEI−2.5 ANPI	7.3	Ni^{2+}	1000	98.1	IP	[2]
PA-PDMC	13.8	Cu^{2+}	100	93.5	IP	[3]
PEI@β-CD/TFNc	7.8	Cr^{3+}	1000	99.8	IP	[4]

PEI-TMC	3.4	Ni^{2+}	1000	96.3	IP	[5]
M6(ED-g-	8.1	Ni^{2+}	1000	90.7	NIPS	[6]
MWCNT)/		Cu^{2+}	1000	91.9		
PES						
DPC-TFC	3.7	Cr^{3+}	1000	98	IP	[7]
		Ni^{2+}	1000	95.8		
G-TFC	5.4	Ni^{2+}	500	90.2	IP	[8]
SEPNF-	9.2	Cu^{2+}	1000	96.7	SEP	[9]
0.24		Ni^{2+}	1000	97.7		
PAN/SPEB	7.6	Ni^{2+}	1000	>93	IP	[10]
-TFC						
TFCM@50	8.2	Cu^{2+}	100	93.9	TAIP	[11]
M2	9.6	Co^{2+}	50	89.6	NIPS	[12]
		Cu^{2+}	50	86.6		
(PEI@GA/	1.9	Cr^{3+}	500	91.8	LBL	[13]
NO-CMC)		Ni^{2+}	500	90.7		
1.5/PES		Cu^{2+}	500	87.2		
6-$PEI_{1.0}Na$/	1.6	Ni^{2+}	200	>95	LBL	[14]
$LS_{1.0}Na$		Cu^{2+}	200	>95		
		Ni^{2+}	200	>95		
		Mn^{2+}	200	>95		
GO-10	4.7	Ni^{2+}	1000	98.9	LBL	[15]
CHIT-DMS	3.2	Co^{2+}	20	98	LBL	[16]
$N-NH_2$		Cu^{2+}	20	97		
		Ni^{2+}	20	93		
DL	6.8	Cu^{2+}	0.64	90	-	[17]
DK	3.2	Cu^{2+}	0.64	97	-	[17]
NF270	13.9	Ni^{2+}	5	70.1	-	[18]
		Cu^{2+}	5	74		
NF90	2.10	Ni^{2+}	200	97	-	[19]
		Cu^{2+}	200	97		
PSS/$T_{0.5}$-	6.4	Co^{2+}	500	96.1	LBL	This
PEI		Cr^{3+}	500	99.7		work
		Ni^{2+}	500	96.0		
		Cu^{2+}	500	92.5		
		Mn^{2+}	500	90.3		

*The abbreviations represent different fabrication methods for nanofiltration membranes: IP (interfacial polymerization), NIPS (nonsolvent-induced phase separation), SEP (sustainable electrospray polymerization), TAIP (thermally activated interfacial polymerization), and LBL (layer-by-layer assembly).

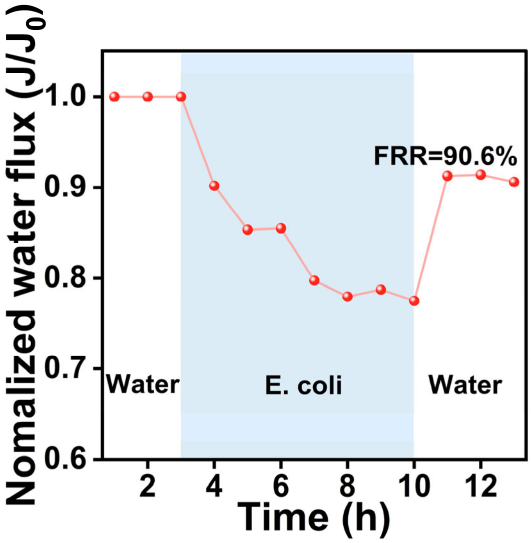

Figure S5. Dynamic antibacterial results for the PSS/$T_{0.5}$-PEI membrane (*E. coli*,10^8 CFU·L^{-1}).

Reference

[1] X Cheng, Y Zhang, S Shao, C Lai, D Wu, J Xu, X Luo, D Xu, H Liang, X Zhu. Highly permeable positively charged nanofiltration membranes with multilayer structures for multiple heavy metal removals. Desalination, 2023, 548: 116266.

[2] Y Bai, P Gao, R Fang, J Cai, L D Zhang, Q Y He, Z H Zhou, S P Sun, X L Cao. Constructing positively charged acid-resistant nanofiltration membranes via surface postgrafting for efficient removal of metal ions from electroplating rinse wastewater. Sep. Purif. Technol., 2022, 297: 121500.

[3] B Wu, X D Weng, N Wang, M J Yin, L Zhang, Q F An. Chlorine-resistant positively charged polyamide nanofiltration membranes for heavy metal ions removal. Sep. Purif. Technol., 2021, 275: 119264.

[4] Y Li, Y Ma, J Dai, Z Ye, L Wu. Nanofiltration membrane with surface nanostructure induced by interfacial modification for heavy metal ions removal, salt selection and antibacterial. J. Environ. Chem. Eng., 2024, 12(2): 112439.

[5] P Xu, W Wang, X Qian, H Wang, C Guo, N Li, Z Xu, K Teng, Z Wang. Positive charged PEI-TMC composite nanofiltration membrane for separation of Li^+ and Mg^{2+} from brine with high Mg^{2+}/Li^+ ratio. Desalination, 2019, 449: 57-68.

[6] M Peydayesh, T Mohammadi, S K Nikouzad. A positively charged composite loose nanofiltration membrane for water purification from heavy metals. J. Membr. Sci., 2020, 611: 118205.
[7] Y Qi, L Zhu, X Shen, A Sotto, C Guo, J Shen. Polythyleneimine-modified original positive charged nanofiltration membrane: removal of heavy metal ions and dyes. Sep. Purif. Technol., 2019, 222: 117-124.
[8] M Li, Z Lv, J Zheng, J Hu, C Jiang, M Ueda, X Zhang, L Wang. Positively charged nanofiltration membrane with dendritic surface for toxic element removal. ACS Sustain. Chem. Eng., 2017, 5(1): 784-792.
[9] Z Ma, L F Ren, D Ying, J Jia, J Shao. Sustainable electrospray polymerization fabrication of thin-film composite polyamide nanofiltration membranes for heavy metal removal. Desalination, 2022, 539: 115952.
[10] T Z Jia, J P Lu, X Y Cheng, Q C Xia, X L Cao, Y Wang, W Xing, S P Sun. Surface enriched sulfonated polyarylene ether benzonitrile (SPEB) that enhances heavy metal removal from polyacrylonitrile (PAN) thin-film composite nanofiltration membranes. J. Membr. Sci., 2019, 580: 214-223.
[11] X Cheng, C Lai, J Li, W Zhou, X Zhu, Z Wang, J Ding, X Zhang, D Wu, H Liang, C Zhao. Toward enhancing desalination and heavy metal removal of TFC nanofiltration membranes: a cost-effective interface temperature-regulated interfacial polymerization. ACS Appl. Mater. Interfaces., 2021, 13(48): 57998-58010.
[12] H Ranjbaran, E Ameri, B Dehghani. Preparation of dendrimer/TiO_2 polysulfone nanofiltration membrane to improve antibacterial, antifouling and separation performance of contaminants (heavy metals, salts, dyes). Polym. Bull., 2024, 81(2): 1471-1494.
[13] C Xiong, Z Huang, Z Ouyang, M Tang, X Lin, Z Zhang. Improvement of the separation and antibiological fouling performance using layer-by-layer self-assembled nanofiltration membranes. J. Coat. Technol. Res., 2020, 17(3): 731-746.
[14] M Y Xie, J Wang, Q Y Wu. Nanofiltration membranes via layer-by-layer assembly and cross-linking of polyethyleneimine/sodium lignosulfonate for heavy metal removal. Chinese. J. Polym. Sci., 2020, 38(9): 965-972.

[15]Y Zhang, S Zhang, J Gao, T S Chung. Layer-by-layer construction of graphene oxide (GO) framework composite membranes for highly efficient heavy metal removal. J. Membr. Sci., 2016, 515: 230-237.
[16]K M Alotaibi, A K Shukla, A A Alshahrani, E M Alzhrani, S S Alotaibi, A Alswieleh. Layer-by-Layer Assembled Nanocomposite Membranes with Functionalized Dendritic Mesoporous Silica Nanoparticles for Selective Heavy Metal Removal. ACS Omega, 2025, 26(10): 28112-28127.
[17]C F Bennani, O M'hiri. Comparative study of the removal of heavy metals by two nanofiltration membranes. Desalination and Water Treatment, 2015, 53(4): 1024-1030.
[18]M Wanjiya, J C Zhang, B Wu, M J Yin , Q F An. Nanofiltration membranes for sustainable removal of heavy metal ions from polluted water: A review and future perspective. Desalination, 2024, 578: 117441.
[19]B Thabo, B J Okoli, S J Modise, S Nelana. Rejection capacity of nanofiltration membranes for nickel, copper, silver and palladium at various oxidation states. Membranes, 2021, 11(9): 653.
